# Deciphering the hormone regulatory mechanisms of storage root initiation in sweet potato: challenges and future prospects

**DOI:** 10.1093/aobpla/plad027

**Published:** 2023-05-22

**Authors:** Sarah R Mathura

**Affiliations:** The Department of Life Sciences, Faculty of Science and Technology, The University of the West Indies, St. Augustine, Trinidad and Tobago

**Keywords:** Hormone, initiation, *Ipomoea batatas*, sweet potato

## Abstract

Sweet potato (*Ipomoea batatas*) is an economically important food crop that is grown primarily for its edible storage roots. Several researchers have consequently been conducting studies to increase sweet potato yield, and an important aspect of this research involves understanding how storage root initiation occurs. Although significant progress has been made, several challenges associated with studying this crop have resulted in lagging progress compared to other crops and thus sweet potato storage root initiation is not clearly understood. This article highlights the most important aspects of the hormone signalling processes during storage root initiation that needs to be investigated further and suggests candidate genes that should be prioritized for further study, based on their importance in storage organ formation in other crops. Lastly, ways of overcoming the challenges associated with studying this crop are suggested.

## Teaser

This article reviews the current understanding of the hormone signalling pathways involved in the initiation of the growth of sweet potatoes. It highlights the gaps in the current knowledge of genes involved in the process and suggests future directions to overcome the challenges associated with studying sweet potatoes. This is important for researchers trying to improve sweet potato yields.

## Introduction

Tubers and storage roots (SRs) form a significant contribution to the world’s food supply. Among these crops, sweet potato (*Ipomoea batatas*) has been ranked sixth in importance (The International Potato Center [CIP] 2017). This crop is considered an important staple that provides a significant portion of energy in the diets of many people who live in developing countries (Titus and Lawrence 2015). Additionally, the sweet potato tuber has high nutritional and medicinal values (Kareem 2013). Due to the economic importance of sweet potato, decades of research have been conducted to investigate how this crop tuberizes to improve yields. A brief summary of the available literature indicates that the anatomical changes during tuber development, the hormones involved in tuberization and changes in gene expression have been widely studied. Some of the genes and molecular processes involved in carbohydrate metabolism, lignification, storage protein synthesis and transcription factor (TF) synthesis have previously been identified.

Despite the previous research that has been conducted to elucidate the mechanism of sweet potato tuberization, the current information is fragmentary, so the entire molecular mechanisms that control tuberization are still unclear due to difficulties in studying this crop (Tanaka 2016; Singh *et al.* 2019). Due to its complex, hexaploid genome, there has been slower progress in understanding the genetic and molecular basis of tuberization in this crop (Yan *et al.* 2022) when compared to more completely sequenced and similar plant species such as potato. Currently, breeding programmes can take years to produce a new cultivar, starting from millions of seeds and is thus expensive and labour intensive. Therefore, to breed new sweet potato varieties with improved yield, the molecular mechanisms for the events involved in tuberization must be clearly defined.

Several questions remain unanswered, such as whether there is a special signal(s) that initiates tuberization, if the mechanism is similar to that in better-studied tuber crops (such as potato) and how hormones regulate tuberization at the molecular level. If these questions can be answered, this would be useful for researchers to identify what genetically affects sweet potato yield and thus develop better-yielding varieties faster by selecting new biomarkers, than what is presently available.

Previous reviews (Ravi *et al.* 2014; Zierer *et al.* 2021; Yang *et al.* 2023) have provided an overview of the hormonal regulation of SR initiation in sweet potato and will not be discussed here. This review seeks to summarize the most recent research on the downstream effectors of hormone signalling pathways that coordinate hormone-mediated responses in sweet potato SR initiation and identify possible further effectors that were studied in other tuberizing crops (e.g. potato and cassava). This article seeks to highlight key areas that are poorly understood but are critical for understanding sweet potato storage organ initiation and suggests important candidate genes that should be studied further. Various strategies for tackling these research problems are also highlighted.

The information provided in this review can be used to prioritize the best genes/proteins that can be studied in greater depth to understand the complex pathways that are involved in hormonal crosstalk during SR initiation. Additionally, this article identifies gaps that should be investigated in the future.

## Anatomical Changes During Storage Root Initiation or Introduction

There are currently two competing hypotheses on the developmental origins of SRs (Carluccio *et al.* 2022). The first, and most widely held hypothesis, is that a developmental change occurs in a subset of fibrous roots (FRs) to yield SRs, and thus SR yield is directly related to this developmental transition. A more recent competing hypothesis states that the developmental fate of the FRs is already determined by their formation and emergence.

The anatomical changes that occur during SR formation have been well documented previously in several reviews and are only briefly described here. During SR initiation, the polarity of root growth changes from longitudinal to radial (Indira and Kurian 1973) and the SR stores starch grains through localized lateral bulking in a sub-apical region of the thick adventitious roots (Ravi *et al.* 2009). In the initial stages of SR development, there is the formation of vascular cambia as primary vascular cambium initials are first laid down within the parenchymatous zone between the protophloem and protoxylem of young FRs (Tanaka 2016). These vascular cambiums become continuous through the division of the pericycle (Ravi *et al.* 2009), forming a circular primary vascular cambium (Tanaka 2016). Further activity of the vascular cambia causes centripetal proliferation of storage parenchyma, secondary vascular tissues and a regular cylinder of vascular cambium (Ravi *et al.* 2009).

Subsequently, there is anomalous secondary growth in which anomalous primary and secondary cambia are formed (Indira and Kurian 1973). Secondary meristems, such as anomalous circular secondary cambia and meristems that surround vessels, differentiate in the xylem (Tanaka 2016). This second cambium is eventually succeeded by a third layer of cambium that starts at the outer limit of the phloem (Indira and Kurian 1973). This process is repeated several times, finally culminating in the formation of concentric zones of alternating xylem and phloem, with the phloem forming most of the storage tissue. An expansion of root diameter is the consequence of cell divisions and expansions in the secondary meristems and primary cambium, as well as divisions of large parenchyma cells that are located in the xylem (Tanaka 2016). During the period of growth due to secondary thickening, the procambial ring progressively aligns outwardly, while the parenchymal cells inside become extremely proliferated and eventually differentiate into thin-walled, starch-storing cells (Ku *et al.* 2008). This leads to the thickening of the SRs and suppression of lignification of the stele (Ravi *et al.* 2009). Starch is mainly stored in the cortex but can also be found in the vascular region (Indira and Kurian 1973).

## Hormonal Control of Tuberization Initiation

Inputs from hormone signals are driving factors for organogenesis (Ku *et al.* 2008). The internal balance of plant hormones is vital to the phase transition and tuber development (Ku *et al.* 2008). It has been hypothesized that plant hormones such as cytokinins (CKs), auxin, jasmonic acid (JA) and abscisic acid (ABA) have various roles in the initiation and thickening processes of the SRs (Wang *et al.* 2015). However, the distinct roles of each hormone in the process are yet to be directly elucidated (Wang *et al.* 2015).

## Auxin

Auxin levels were observed to be high during the initial stages of SR formation (Noh *et al.* 2013), but these levels decline as secondary thickening growth progresses (Dong *et al.* 2019). Studies using transgenic potato plants revealed that overexpression of the auxin biosynthetic genes, *AtYUCCA6* and *StYUCCA8*, led to reduced tuber yield (Kim *et al.* 2013; Roumeliotis *et al.* 2022). Evidently, this regulation of auxin concentration throughout storage organ development is crucial. However, it is currently unclear how feedback mechanisms result in the auxin concentration declining after SR initiation. Studies using overexpression of auxin biosynthetic genes in sweet potato can contribute towards a better understanding of which hormonal and transcriptional pathways are affected when auxin is present in excess.

Vascular cambium formation in SRs is thought to be triggered by auxin in a way that is similar to that of *Arabidopsis* and poplar (Zierer *et al.* 2021). In poplar, PIN-FORMED (PIN) proteins mediate polar auxin transport from the shoot to the root, forming a gradient of auxin concentration that peaks in the vascular cambium (Zierer *et al.* 2021). This gradient is hypothesized to send spatial signals to cambial stem cells, with high concentrations causing cell division, intermediate concentrations leading to cell expansion and low concentrations triggering secondary wall formation (Zierer *et al.* 2021). In potato, tuberization is stimulated by the mobile RNAs *StSP6A* and *StBEL5*, which induce the expression of the tuber marker genes, *StPIN1*, *StPIN*, and *StPIN4* (Sharma *et al.* 2016). It is currently unknown whether these *PIN* genes are also crucial for sweet potato SR formation and if so, whether they interact with these mobile RNAs. Genome-wide identification of the *IbPINs* and their expression at SR initiation are necessary to determine which PINs are involved in this process.

Auxin exerts its effects via the auxin signalling pathway which includes the auxin response factor (ARF) TFs. Auxin response factors are upregulated during SR initiation in potato and cassava (Utsumi *et al.* 2022). Since *StARF8* is a tuber marker gene that is targeted by *StBEL5* (Sharma *et al.* 2016), *IbARF8* should be explored or characterized in sweet potato and its transcriptional targets. This gene is likely to be very important for tuberization, as the *Ipomoea trifida* homolog, *ItfARF8*, is strongly expressed in sweet potato SRs (Pratt and Zhang 2021). Additionally, Kang *et al.* (2018) found that *IbARF5* positively regulated carotenoid biosynthesis and drought and stress tolerance. However, *in silico* analyses indicate that *IbARF5* is downregulated in SRs versus FRs (Mathura *et al.* 2023).

Other components of the auxin signalling pathway include the auxin/indole-3-acetic acid (Aux/IAA) proteins (that inhibit ARFs at low auxin concentrations), Small auxin upregulated proteins (which are effectors of hormone signals) and Gretchen-Hagen 3 proteins (which inactivate auxin). Several studies have shown that some members of these gene families are differentially expressed during storage organ formation in potato, cassava and sweet potato (Gao *et al.* 2016; Ding *et al.* 2020; Rüscher *et al.* 2021; Utsumi *et al.* 2022; *et al.*Mathura *et al.* 2023). It is, therefore, necessary to perform functional validation studies with these genes to elucidate their specific roles during the storage organ formation.

## Cytokinins Are Involved in the Activity of the Primary Cambium

Several studies have shown that CKs are likely to have crucial roles in the initiation and development of sweet potato SRs (Ku *et al.* 2008; Dong *et al.* 2019). Zeatin (Z) is the most active CK in higher plants and exists in the forms *cis*-zeatin (*c*Z), *trans*-zeatin (*t*Z), *cis*-zeatin riboside (*c*ZR) and *trans*-zeatin riboside (*t*ZR) (Kieber and Schaller 2018). Other active forms of CK include dihydro-zeatin, dihydro-zeatin riboside, isopentenyl (iP) and isopentenyl adenine (Wang *et al.* 2006). CK levels are relatively high during the initial stages of SR formation (Noh *et al.* 2013) and different studies have shown that CK content increases as the SR matures (Wang *et al.* 2006; Dong *et al.* 2019). In cassava, iP and *t*Z are elevated at SR initiation, while *c*Z is high in pre-tuberous roots (Utsumi *et al.* 2022).

The endogenous CK, *t*ZR, was proposed to have vital roles in SR initiation by developing and activating the primary cambium (Tanaka *et al.* 2005). The level of endogenous CK in sweet potato roots has been observed to quickly increase at the start of SR formation (Tanaka 2016). There has also been a report of exogenous applications of CK enhancing SR formation (Tanaka 2016). However, it seems that high levels of endogenous sucrose must also be present for CK to induce SR initiation (Eguchi and Yoshida 2008). CKs have been shown to be important regulators of cambial activity in *Arabidopsis*, so it is likely that CKs have crucial roles in the formation of the SR via regulation of activity of the primary cambium (Tanaka 2016).

There is evidence that supports a link between the expression of Knotted-like Homeobox (*KNOX*) genes and hormone signalling pathways (Ravi *et al.* 2014). There are two subclasses of *KNOX* genes—Class I and Class II, and several *KNOXI* genes have been reported to have roles in sweet potato SR formation (Ravi *et al.* 2014). *KNOXI* genes have been reported to regulate SR development and CK levels in sweet potato SRs (Ravi *et al.* 2014). Tanaka *et al.* (2008) isolated three *KNOXI* genes named *Ibkn1*, *Ibkn2* and *Ibkn3*. The expression of these genes in mature and developing SRs was higher than in FRs, where the expression of these genes was low or undetectable (Tanaka *et al.* 2008). The distribution of *t*ZR in the SRs was similar to the pattern of expression of these *KNOXI* genes, which suggests that there is a link between the expression of *KNOXI* genes and CK levels in SRs (Tanaka *et al.* 2008). The preferential expression of *Ibkn1* around the primary vascular cambium suggests that the Ibkn1 protein functions in the maintenance of the indeterminate state of the cells in this region (Tanaka *et al.* 2008).

*KNOXI* genes are proposed to be involved in the regulation of CK levels in several plants since *KNOXI-*overexpressing plants show elevated CK levels (Ravi *et al.* 2014). In sweet potato, there may be a correlation between the high expression of *KNOXI* genes and the elevated expression of *t*ZR in developing SRs (Ravi *et al.* 2014). There are lower *t*ZR levels in the distal end of the SR, where there is also decreased *KNOXI* gene expression (Ravi *et al.* 2014). However, the possibility that CKs modulate the expression of *KNOXI* genes cannot be ignored. Based on the observations in other plants, it is possible that KNOX proteins orchestrate growth-regulation homeostasis by repressing gibberellic acid (GA) and upregulating CKs (Zierer *et al.* 2021). Additionally, CKs may also be responsible for stimulating the storage metabolism of the proliferating parenchyma cells (Zierer *et al.* 2021).

While CK is necessary for SR formation, it cannot exert these effects without auxin. In cassava, it is suggested that the auxin-to-CK ratio is crucial for SR initiation, so this level must be tightly controlled (Utsumi *et al.* 2022). It is possible that after the committed step of SR initiation occurs, a negative feedback mechanism allows the auxin level to decline and the CK level to increase.

## Gibberellins Promote Lignification Which Inhibits SR Formation

Gibberellic acid promotes lignification, which is a process that is downregulated in SRs and thus GA levels decline as storage organ formation progresses (Singh *et al.* 2019). The exact mechanisms by which GA negatively regulates tuberization and how its levels decline as SR development progresses are currently unclear. DELLA proteins are core components of the GA signalling pathway and experimental evidence suggests a role for DELLA-dependent signalling pathways in yam and potato tuber growth (Chen *et al.* 2022a). DELLA proteins prevent transcription of genes whose expression can be modulated by GA. The presence of GA leads to the ubiquitination and degradation of DELLAs, thus facilitating transcriptional activation of these genes. It is, therefore, possible that genes involved in lignification are directly activated by GA, so that lowered GA levels lead to transcriptional repression. In *Arabidopsis thaliana*, the GA biosynthetic gene, *GA 20-oxidase* (*GA20ox*), is a target of DELLA (Chen *et al.* 2022a), so is it possible that DELLA negatively regulates GA biosynthesis during SR formation?

Another way in which DELLA signalling may contribute towards tuberization can be through its interaction with genes involved in circadian rhythm and flowering time regulation, such as *CONSTANS*, *PHYTOCHROME INTERACTING FACTOR 4 (PIF4)* and *FLOWERING LOCUS T* suppressors (Chen *et al.* 2022a). These genes have been investigated in potato, but there is an urgent need to investigate whether these genes are crucial for sweet potato SR initiation and, if so, whether they interact with DELLAs to mediate their effects.

## Abscisic Acid Is Involved in Expansion of the Developing SR

Abscisic acid is likely to be involved in both SR initiation (Dong *et al.* 2019) and expansion (Wang *et al.* 2006). Like auxin, ABA concentration peaks at SR initiation and declines during SR expansion (Dong *et al.* 2019). Abscisic acid is mostly associated with cell differentiation in the vascular cambium during the later developmental stages (Ku *et al.* 2008). Abscisic acids have been proposed to regulate the thickening of the SR by activating cell division at the meristem, particularly at the secondary meristem in the xylem that is located on the inside of the primary cambium (Tanaka *et al.* 2005). The observation was also made that endogenous ABA localizes around the primary cambium and meristem in the xylem (Tanaka *et al.* 2005). Abscisic acid regulates cell differentiation and SR thickening either on its own or via interactions with CK (Ravi *et al.* 2014). Thus, the internal balance of ABA to CK may be vital for SR development (Ravi *et al.* 2014).

Abscisic acid also promotes the distribution and deposition of carbohydrate through the SR by promoting starch synthase activity (i.e. increased starch synthesis; Wang *et al.* 2006; López-González *et al.* 2019). Based on the findings in other tuber crops, ABA can also promote the activity of the starch-synthesizing enzyme, ADP–glucose pyrophosphorylase (AGPase), but decrease the activity of the starch-degrading enzymes, α-amylase and β-amylase (López-González *et al.* 2019).

It has been suggested that ABA and GA have antagonistic effects during the development of stem and root tubers in many crops, with a higher ABA:GA ratio promoting tuberization and a lower ratio inhibiting tuberization (Chen *et al.* 2022a). The ABA signalling pathway has the key components: pyrabactin resistance-like proteins, protein phosphatases of type 2Cs, sucrose non-fermenting-1-related protein kinase 2s, and ABA-responsive element-binding proteins/factors. Some members of this pathway, such as *StABF2* and *StABF4*, have been implicated in tuber formation and yield (Dutt *et al.* 2017). Identification of the corresponding genes in sweet potato would be important as a first step towards investigating whether these genes are regulated by DELLA and integrate the ABA and GA signalling pathways.

## Jasmonic Acid and SR Expansion

Jasmonic acid affects root pigmentation which serves as an early initializing signal for formation of the root tuber (Ku *et al.* 2008). Jasmonic acid has been shown to suppress root elongation in some plants and this may also be applicable in sweet potato since the process of SR formation involves the cessation of longitudinal root growth in favour of radial root growth (Noh *et al.* 2013). Exogenous JA application increased the probability of SR formation and led to an increase in SR diameter by increasing the number of cells in the cortex (Nakatani 1994). Jasmonic acid levels show a progressive decline as SR development progresses, with an eventual increase as the SR reaches maturity (Dong *et al.* 2019). Huang *et al.* (2021) investigated the expression of genes encoding the jasmonate ZIM-domain (JAZ) protein family which are involved in JA signalling. *IbJAZ3.1*, *IbJAZ4*, *IbJAZ6.1*, *IbJAZ8.2*, and *IbJAZ9* were expressed significantly higher in SRs compared to FRs, but these genes were also highly expressed in other plant parts as well. The expression of these genes was repressed by ABA and showed varying responses to GA, IAA, MeJA and SA (Huang *et al.* 2021). Overexpression of the B-box TR, *IbBBX24*, led to increased sweet potato yield since *IbBBX24* interacts with *IbJAZ10* reducing *IbMYC2* repression and increasing transcription of genes that are transcriptionally activated by *IbMYC2* (Zhang *et al.* 2020). Therefore, there are multiple lines of evidence to suggest that JA signalling is important for SR formation, but further experimental evidence is required to decipher the mechanisms by which this occurs.

## Ethylene and SR Initiation

Several genes that are involved in ethylene signalling pathways (which include AP2/EREBP (ethylene-responsive element-binding protein) TR, callus-expressing factor, ethylene response factor 4, AP2/EREBP TR ERF-2, dehydration-responsive element-binding protein and ethylene-responsive TR 3) were found to be upregulated in FRs but not SRs (Desai 2008). However, the TR DREB1A, which is involved in ethylene signalling, and 1-aminocyclopropane-1-carboxylate oxidase, which is involved in ethylene synthesis, were upregulated in SRs but not FRs (Desai 2008). These results indicate that ethylene signalling pathways could have roles during SR initiation, but it is unclear whether ethylene is a major hormonal regulator of SR formation.

## Brassinosteroids (Positively Regulate SR Initiation

Brassinosteroids (BRs) are involved in various aspects of plant development, including root growth, thermotolerance, drought tolerance, and light responses (Lv and Li 2020). Some of these responses are mediated by the antagonistic effects of ABA and BR (Lv and Li 2020). The storage phase has been suggested to be modulated by BR signalling pathways since there was observed upregulation in the storage phase of genes that function in brassinolide synthesis (steroid 5 alpha-reductase) and brassinolide signalling (leucine-rich repeat family protein-like; Desai 2008). In cassava, experimental evidence suggests that BR positively affects SR formation by modulating auxin signalling (Utsumi *et al.* 2022). It is interesting to note that BR signalling restricts lignification in *A. thaliana* roots (Li *et al.* 2022), so it would be worthy to investigate whether there is downregulated expression of genes involved in lignification in BR-overexpressing sweet potato plants. To distinguish the potential roles of BRs in SR initiation from their roles in general plant development, the expression of the genes involved in the core BR signalling pathway should be mined to identify whether any are involved in SR initiation. Additionally, the BR concentration throughout SR development should be quantified in sweet potato, as performed by Dong *et al.* (2019) for the other hormones discussed earlier.

## Salicylic Acid May Not Be Directly Involved in SR Initiation

It is not likely that SA has any major role in the SR initiation process. Utsumi *et al.* (2022) did not find any significant effect of SA on cassava tuberization. Additionally, SA was not detected in potato tubers naturally *in vivo*, even though SA can promote potato tuberization *in vitro* (Koda *et al.* 1992).

## Crosstalk Between Hormone Signalling Pathways

These plant phytohormones work synergistically to mediate SR initiation. While the details of this crosstalk are yet to be described, two important points warrant further investigation. First, how do these hormones integrate signals from the environment to trigger SR initiation? Hormones act as messengers, which respond to the various environmental cues that facilitate SR initiation and relay these signals to various TFs and miRNAs to regulate the transcription of downstream genes. Conditions such as the absence of light, adequate supply of nutrients and water, and sufficient oxygen levels must be sensed by the plant. Target of Rapamycin (TOR) signalling is a central hub that integrates environmental signals with hormone and nutrient signalling (Fu *et al.* 2020). Target of Rapamycin signalling has already been linked to BR and sugar signalling, as well as ABA and auxin signalling (Fu *et al.* 2020). Future experimental studies should seek to determine if and how TOR signalling coordinates these hormone responses synergistically in SR development.

Another line of investigation is the presence of multiple hubs or pathways. Are there multiple hubs and pathways that lead to SR initiation? Hannapel (2013) suggested that since potato storage organ formation is a huge investment in nutrient resources and energy, there are overlapping pathways that can act as backups to endure that storage organ formation is successful. Given that members of various hormone signalling pathways are co-expressed with hub genes, this hypothesis is plausible in sweet potato as well.

## Challenges to Elucidating the Complete Tuber Initiation Pathway and Proposed Solutions

While a variety of experimental protocols have been developed to investigate the complex regulatory mechanisms of hormone signalling in plants, many of these techniques are currently very costly, and difficult or simply unfeasible to apply without further improvements. One such technique is chromatin immunoprecipitation sequencing (ChIP-seq), which identifies TF-binding targets. However, this technique is very challenging to perform, thus making it prone to experimental error and artefacts (Xie *et al.* 2020). Despite these barriers, there is one ChIP-seq experiment (NCBI accession PRJNA589088) performed on sweet potato in response to disease to date (Zhang *et al.* 2020). Future improvements to this technique would lead to ChIP-seq being more widely adopted in sweet potato research to better understand the epigenetic regulation of SR initiation. For example, Class A ARF TFs possibly involved in SR initiation are transcriptional activators, and one of the mechanisms by which they exert their effects is through interactions with chromatin remodelling proteins, to enhance transcription of their downstream targets. Identification of these downstream targets would be important to link auxin signalling with epigenetic transcriptional regulation.

Another one of these techniques is single-cell RNA-seq (scRNA-seq). The ability to analyse transcriptomic data to the resolution of different cell types would be indispensable to determine how different cellular fates arise, particularly that of the adventitious roots at emergence which can lead to FRs or SRs. However, the application of this technique is limited in plants due to the presence of the cell wall preventing the dissociation of tissues into individual cells during protoplast formation (Xie *et al.* 2020). Thus, this technique is yet to be successfully reported in sweet potato, although it has been done in a few model crops. In the future, new and promising techniques are expected to overcome this hurdle. Long *et al.* (2021) successfully created a protoplast-free protocol, called flsnRNA-seq, although they note that this technique is limited by the difficulty in isolating nuclei from certain plants.

There have also been recent developments in the use of mass spectrometry (MS) to determine plant phytohormone levels at various stages of development and map phytohormone-dependent protein interactions (Chen *et al.* 2022b). The use of high-throughput proteomic data to complement the current transcriptomic era of sweet potato research is important. While further development of computational tools to analyse this data and enhanced wet-lab protocols for metabolite extraction are needed, MS is a promising approach. A protocol for analysing the sweet potato proteome via MS has already been reported (Al-Mohanna *et al.* 2019).

Another challenge to investigating SR initiation in sweet potato is the difficulty associated with growing SRs *in vitro*, leading to a paucity of reports of *in vitro* grown SRs. This is in contrast to potato, where there are more established culturing protocols in place to investigate tuberization. These culturing systems would allow the effects of exogenous hormone application to be easily quantified (without the environmental variability of field experiments) by measuring the changes in the expression of important genes.

As previously mentioned, the availability of an accurate reference genome is vital for the continued progress in this field. The most recent version of the genome was published in 2022 by the SweetGAINS Project (http://sweetpotato.uga.edu/sweetgains_beauregard_v1_asm_anno.shtml). This genome, along with better versions in the future, will facilitate more genome-wide analyses of gene families to understand their roles in SR initiation. Transcription factors belong to large gene families, such as the MADS-box gene family, which has been widely studied to understand their importance in SR formation (Kim *et al.* 2005; Ku *et al.* 2008; Dong *et al.* 2018). Additionally, there is a need for better curating and annotation of genes that have been well characterized onto these newly released genomes. Research teams should develop a curated database with expression data readily available for the hypothesis-free identification of genes that are involved in storage organ formation. The expression data displayed on the SweetGAINS genome assembly are for sweet potato SR and FR samples at different stages of development and during abiotic stress using Beauregard and Tanzania cultivars. It is expected that future genome assemblies will include data from several other cultivars worldwide, just as there are curated expression atlases for model crops. Network analysis using results from thousands of available transcriptomic datasets have been used to find key hub genes involved in various plant developmental processes using Weighted Gene Coexpression Network Analysis. He *et al.* (2021) used Dynamic Network Biomarker analysis to find genes responsible for triggering sweet potato SR initiation and found *IbNAC083* to be involved in this process. These network analysis methods will be useful for reducing the search space of potential gene candidates for further study via loss/gain-of-function experiments.

Another possibility could be the creation of a curated database of known tuberization pathways for crops of economic importance. There is a wealth of information on tuberization in other crops, of which some should remain consistent with the mechanism in sweet potato. While this article summarizes some of the key findings from other crops that can be applied to sweet potato, the exponential increase in results from such publications may best be categorized in a curated pathway database.

Various transcriptomic approaches have been conducted to identify differentially expressed genes (DEGs) between tuberizing and non-tuberizing sweet potato tissue and these DEGs have given invaluable insight into which processes are regulated and how they are regulated during tuberization. These comparisons include SR versus FR (Firon *et al.* 2013), distal SR end versus proximal SR end (Singh *et al.* 2020), and *I. batatas* SR versus *I. trifida* roots, with the former in each comparison being analogous to the tuberizing state and the latter representing the non-tuberizing tissue state. It was observed by Sirju-Charran and Wickham (1988) that the buried underground stem, when planted in an inverse orientation, can tuberize and become an alternative storage site. Currently, we are conducting research that compares the transcriptome of tuberizing versus non-tuberizing underground sweet potato stems in order to further understand the tuberization initiation process.

The experimental suggestions described above will complement the existing studies conducted on individual genes to determine their roles in SR formation.

## Conclusion

Sweet potato SR initiation is a complex process that involves the interplay of several signalling pathways that involve various hormones, TFs and miRNAs. Prior to the release of a high-quality genome reference, progress in elucidating these pathways lagged in comparison to other well-studied storage organs, such as potato and cassava. It is expected that sweet potato research will accelerate with the availability of improved databases and experimental resources. While the present era of research on SR formation in sweet potato is largely based on transcriptomic data, it is expected that more proteomic and metabolomic data will complement these studies. This will lead to the improvement in our knowledge on these complex regulatory pathways and this should translate into producing better-yielding sweet potato varieties.

## Data Availability

No new data were generated or analysed in support of this research.

## Notes

**Phenome, Genome and Environment. Chief Editor:** Colleen Doherty
